# Attenuation of Nicotine Effects on A549 Lung Cancer Cells by Synthetic α7 nAChR Antagonists APS7-2 and APS8-2

**DOI:** 10.3390/md22040147

**Published:** 2024-03-26

**Authors:** Ahmad Joukhan, Veno Kononenko, Tadeja Bele, Marija Sollner Dolenc, Steve Peigneur, Ernesto Lopes Pinheiro-Junior, Jan Tytgat, Tom Turk, Igor Križaj, Damjana Drobne

**Affiliations:** 1 Department of Pharmaceutical Chemistry, Faculty of Pharmacy, University of Ljubljana, 1000 Ljubljana, Slovenia; marija.sollner@ffa.uni-lj.si; 2 Department of Biology, Biotechnical Faculty, University of Ljubljana, 1000 Ljubljana, Slovenia; veno.kononenko@bf.uni-lj.si (V.K.); tom.turk@bf.uni-lj.si (T.T.); 3 Department of Molecular and Biomedical Sciences, Jožef Stefan Institute, 1000 Ljubljana, Slovenia; tadeja.bele@gmail.com (T.B.); igor.krizaj@ijs.si (I.K.); 4 Faculty of Medicine, University of Ljubljana, 1000 Ljubljana, Slovenia; 5 Laboratory of Toxicology and Pharmacology, KU Leuven, 3000 Leuven, Belgium; steve.peigneur@kuleuven.be (S.P.); ernestolopesjr@gmail.com (E.L.P.-J.); jan.tytgat@kuleuven.be (J.T.)

**Keywords:** marine toxin, nicotinic acetylcholine receptor, nAChR antagonist, APS7-2, APS8-2

## Abstract

Nicotine binds to nicotinic acetylcholine receptors (nAChRs) that are overexpressed in different cancer cells, promoting tumor growth and resistance to chemotherapy. In this study, we aimed to investigate the potential of APS7-2 and APS8-2, synthetic analogs of a marine sponge toxin, to inhibit nicotine-mediated effects on A549 human lung cancer cells. Our electrophysiological measurements confirmed that APS7-2 and APS8-2 act as α7 nAChR antagonists. APS8-2 showed no cytotoxicity in A549 cells, while APS7-2 showed concentration-dependent cytotoxicity in A549 cells. The different cytotoxic responses of APS7-2 and APS8-2 emphasize the importance of the chemical structure in determining their cytotoxicity on cancer cells. Nicotine-mediated effects include increased cell viability and proliferation, elevated intracellular calcium levels, and reduced cisplatin-induced cytotoxicity and reactive oxygen species production (ROS) in A549 cells. These effects of nicotine were effectively attenuated by APS8-2, whereas APS7-2 was less effective. Our results suggest that APS8-2 is a promising new therapeutic agent in the chemotherapy of lung cancer.

## 1. Introduction

Lung cancer is a major global health concern, being the second most common cancer and leading cause of cancer-related death worldwide [1]. Centers for Disease Control and Prevention (2022) reported that tobacco smoking is a major risk factor for lung cancer, accounting for 80% to 90% of all lung cancer deaths [2]. Lung cancer is classified into two main types: small-cell lung cancer (SCLC) and non-small-cell lung cancer (NSCLC). NSCLC is the most common cancer subtype, accounting for approximately 85%, while SCLC constitutes 15% of all lung cancer cases [3,4]. Treatment of NSCLC is challenging, and often traditional chemotherapy alone does not produce successful outcomes [3,4]. Despite the advancements made in the field of cancer biology, cancer treatment remains a significant challenge. The main problems are indiscriminate action of the drug on healthy cells and cancer cell resistance to chemotherapy [5]. For instance, cisplatin, a commonly used chemotherapy drug in the treatment of lung cancer, often leads to resistance [6,7]. This calls for new treatment strategies that may improve the already existing chemotherapy treatments.

Tobacco smoke is the main risk factor for lung cancer [8], and people who smoke cigarettes are 15 to 30 times more likely to get lung cancer and die from it as compared to non-smokers [2]. Tobacco smoke is a mixture of more than 5000 chemicals, and many of those are considered carcinogens [9], while nicotine represents the addictive component [10]. Nicotine itself has not been proven to initiate tumorigenesis in human cells; however, it promotes tumor growth and metastases [10]. It binds and activates membrane nicotinic acetylcholine receptors (nAChRs), which are overexpressed in numerous cancer types [11]. Activation of α7 nAChRs triggers various signaling pathways that promote the invasiveness of lung cancer cells and block apoptosis, making cancer cells resistant to chemotherapy [12]. This is a matter of significant concern, given the rising use of nicotine attributed to alternative smoking devices, often perceived as a safer alternative to traditional tobacco smoking.

As is well known, nicotine binds to ionotropic nAChRs, which consist of five subunits. nAChRs are divided into homopentameric receptors composed of five α7 or α9 subunits and heteromeric receptors composed of different subunits, such as α2–α6 and β2–β4 subunits [13]. Different subtypes of nAChRs regulate different functions and permit different cations (e.g., Na^+^ or Ca^2+^); the homopentameric α7 nAChRs subtype is particularly known for its high Ca^2+^ permeability [14]. The activation of α7 nAChRs in lung cancer cells was linked to increased cell proliferation, angiogenesis, migration, metastasis, and neurogenesis while inhibiting apoptosis [15]. Hsu et al. (2020) reported that nicotine exposure to cancer cells leads not only to decreased cell apoptosis but also to increased resistance to cisplatin through α7 nAChRs [12]. Furthermore, it was suggested that α7 nAChRs are strongly expressed only in the early phase of tumorigenesis of NSCLC, allowing rapid cell growth and angiogenesis, while thereafter their expression decreases [16]. Hence, it is reasonable to target this pathway with α7 nAChR antagonists for the treatment of NSCLC, especially in the early stages of the disease.

Marine organisms have emerged as valuable **reservoirs** of compounds exhibiting antitumor properties [11], such as the polymeric 3-alkylpyridinium salts (poly-APS) derived from the marine sponge *Haliclona (Rhizoneira) sarai* [17]. These polymeric poly-APS are compounds composed of 29 monomeric N-butyl-3-butyl pyridinium units, each with a 3-octyl chain [18]. Poly-APS exhibited selective cytotoxicity on NSCLC cells; however, it demonstrated higher toxic effects in experimental animals [19]. Considering the potential use of poly-APS-based synthetic compounds in medicine, such as new chemotherapeutic agents, several 3-alkylpyridinium oligomers and polymers have been synthesized [19]. For example, APS8, a synthetic poly-APS analog, has proven to be a potent antagonist of heterologous α7 nAChRs and to modulate these receptors at low concentrations (1–3 nM), but it is much less effective on the α4β2 nAChR [11]. Zovko et al. (2013) showed that APS8 inhibits lung cancer cell growth and activates apoptosis, whereas APS8 is less cytotoxic to normal lung fibroblasts [11]. The effect of APS8 was also tested in vivo on human pulmonary adenocarcinoma xenografts in immunocompromised mice, where the intratumoral application of APS8 significantly reduced tumor growth [20].

This study aimed to investigate the potential of two additional synthetic poly-APS analogs (APS7-2 and APS8-2) to inhibit nicotine-mediated effects on A549 human lung cancer cells. These nicotine effects include increased cell viability and proliferation, elevated intracellular calcium levels, and reduced cisplatin-induced cytotoxicity and reactive oxygen species production in A549 cells. The study initially assessed the potential of APS7-2 and APS8-2 to block α7 nAChRs using electrophysiological measurements. Additionally, their cytotoxicity was tested on A549 human lung cancer cells using three different assays. The effects of nicotine on lung cancer cells were effectively attenuated by APS8-2, whereas APS7-2 was less effective.

## 2. Results

### 2.1. APS7-2 and APS8-2 Block α7 nAChRs

**Figure 1:**
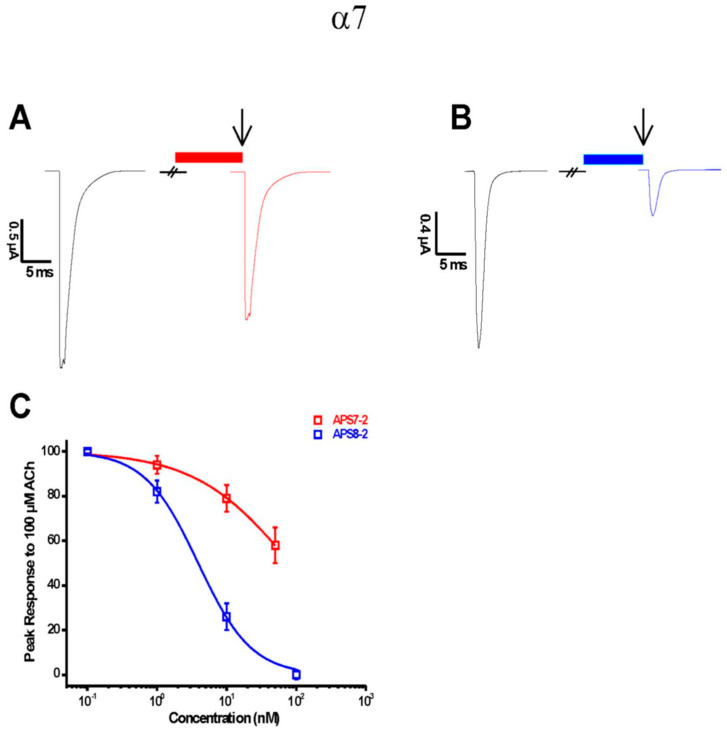
Electrophysiological characterization of APS7-2 and APS8-2 on human heterologous α7 nAChRs expressed in *X. laevis* oocytes. Agonist-evoked responses of human α7 nAChRs using 100 μM acetylcholine (black arrow), first applied alone and then co-applied with (**A**) 10 nMAPS7-2 (red bar) or (**B**) 10 nM APS8-2 (blue bar). (**C**) The data are normalized to the peak amplitude of current recorded without APS (100%) and presented as mean ± SEM (*n* = 5 oocytes).

Electrophysiological measurements were performed on human α7 nAChR receptors heterologously expressed in *Xenopus laevis* oocytes to determine the effects of APS7-2 and APS8-2 on ACh-evoked currents mediated by nAChRs. The results of the tests shown in Figure 1 confirm that APS7-2 and APS8-2 are both capable of inhibiting α7-mediated ACh currents. Concentration–response curves were constructed to determine the IC_50_ (half-maximal inhibitory concentration) values (Figure 1C). The obtained IC_50_ values were 82.4 ± 6.7 nM and 3.9 ± 0.3 nM for APS7-2 and APS8-2, respectively. APS7-2 could not be tested at higher concentrations since cytolysis of the oocytes was observed at 100 nM and above.

### 2.2. Cytotoxicity of APS7-2 and APS8-2 on A549 Lung Cancer Cells

**Figure 2:**
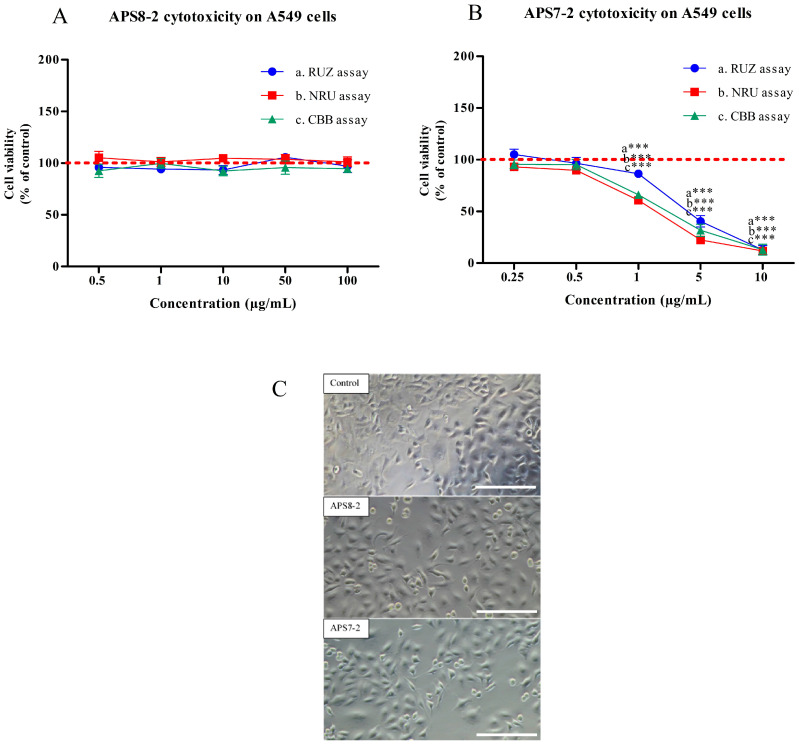
Cytotoxicity of APS8-2 and APS7-2 on A549 human adenocarcinoma lung cancer cells after 24 h exposure. Cytotoxicity was measured using RUZ, NRU, and CBB assays. (**A**) The cytotoxicity of APS8-2 was measured in the range of 0.5 µg/mL to 100 µg/mL, while (**B**) for APS7-2 it was in the range of 0.25 µg/mL to 10 µg/mL. (**C**) Microscopic images (20×) of A549 cells, including control, 0.5 µg/mL APS8-2, and 0.5 µg/mL APS7-2; scale bar represents 100 μm. Measurements were normalized to the untreated control (dashed line) as the mean percentage (±SD). Data were statistically analyzed by ANOVA with Bonferroni multiple comparisons post-test. The asterisks indicate a significant difference compared to the untreated control: *** corresponds to *p* < 0.001.

The cytotoxicity of APS7-2 and APS8-2 was evaluated on A549 lung cancer cells after 24 h exposure using three different assays: resazurin assay (RUZ), neutral red uptake assay (NRU), and Coomassie Brilliant Blue assay (CBB) (Figure 2). APS8-2 showed no cytotoxicity to A549 cells over a wide concentration range (0.5 µg/mL to 100 µg/mL) (Figure 2A), with no changes in metabolic activity (RUZ assay), lysosomal stability (NRU assay), and protein content (CBB assay) observed. In contrast, APS7-2 exhibited concentration-dependent cytotoxicity, with the onset of cytotoxicity observed at a concentration of 1 µg/mL (Figure 2B). Exposure to APS7-2 caused a significant reduction in metabolic activity, lysosomal stability, and protein levels in A549 cells (Figure 2B). A concentration of 0.5 µg/mL APS7-2 and APS8-2 showed no cellular damage in A549 cells (Figure 2C).

### 2.3. Attenuation of Nicotine-Induced Increased Cell Viability and Proliferation by APS8-2 and APS7-2

**Figure 3:**
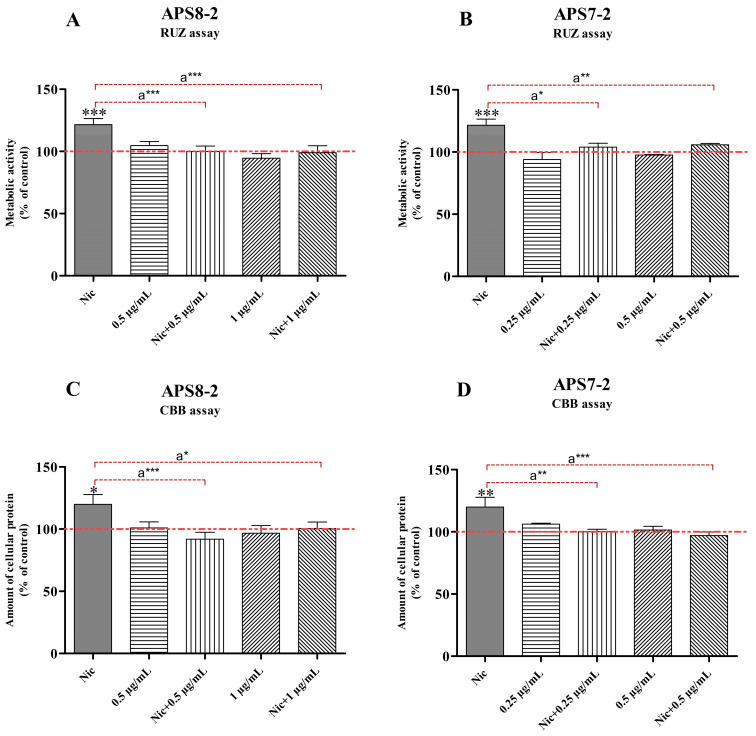
Evaluation of the effects of nicotine (Nic), APS8-2, APS7-2, and the combination of APS8-2 or APS7-2 with nicotine on the viability and proliferation of A549 cells. A549 cell viability and proliferation were assessed using (**A**,**B**) RUZ assay for cell viability and (**C**,**D**) CBB assay for cell proliferation. A549 cells were pretreated with 1 µM nicotine for 24 h, then treated with 0.25 µg/mL APS7-2, 0.5 µg/mL APS7-2, 0.5 µg/mL APS8-2, or 1 µg/mL APS8-2 for another 24 h. Measurements were normalized to the untreated control (dashed line) as the mean percentage (±SD). The data were statistically analyzed by ANOVA with Bonferroni multiple comparisons post-test. Compared with the untreated control (* equals *p* < 0.05; ** equals *p* < 0.01; *** equals *p* < 0.001); compared with nicotine-treated cells (a* equals *p* < 0.05; a** equals *p* < 0.01; a*** equals *p* < 0.001).

A549 cell viability and proliferation were assessed using RUZ and CBB assays. At 1 µM, nicotine increased the viability and proliferation of A549 cancer cells (Figure 3). APS7-2 (Figure 3B,D) and APS8-2 (Figure 3A,C) attenuated the effects of nicotine on the proliferation and viability of A549 cells. However, APS7-2 and APS8-2 caused no changes in cell viability and proliferation in cells not treated with nicotine (Figure 3).

### 2.4. Prevention of Nicotine-Induced Intracellular Ca^2+^ Elevation in A549 Lung Cancer Cells by APS8-2

**Figure 4:**
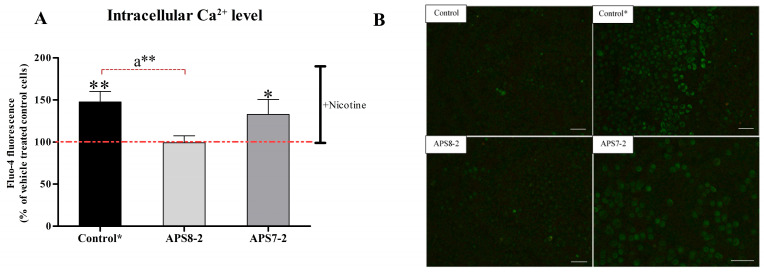
Intracellular Ca^2+^ level in A549 cells after 100 s of adding nicotine. (**A**) Cells were pretreated with APS compounds (1 µg/mL APS7-2 and 1 µg/mL APS8-2) for 24 h in a serum-deprived medium. In the control, cells were not pretreated with APS compounds, and fluorescence was measured after adding 1 mM nicotine. (**B**) Fluo-4 fluorescence intensity was measured in A549 cells. Control was measured after 100 s without adding nicotine. Control* (after adding nicotine) and A549 cells pretreated with APS7-2 and APS8-2 were measured after 100 s of adding nicotine. The scale bar represents 100 μm. The measurements were normalized to untreated cells (control) before adding nicotine, and they are presented as the mean percentage (±SD). Each treatment was conducted two times independently with three replications, and fluorescence data were taken from at least 30 individual cells per repetition. The data were analyzed using ANOVA with multiple comparisons and the Bonferroni post-test, and statistical significance was determined using *p*-values. A comparison was made between the treated cells with nicotine and the untreated control, with * indicating *p* < 0.05 and ** indicating *p* < 0.01. A comparison was also made between the control cells after adding nicotine and those pretreated with APS compounds, with a** indicating *p* < 0.01.

Intracellular calcium levels in A549 cells were measured using the Ca^2+^ indicator Fluo-4 AM to assess the potential of APS7-2 and APS8-2 to prevent nicotine-induced Ca^2+^ influx (Figure 4). After adding 1 mM nicotine to A549 cells, intracellular calcium levels were significantly increased (Figure 4A). However, when the cells were pretreated with 1 µg/mL APS8-2 for 24 h, the nicotine-induced increase in intracellular calcium levels was significantly prevented (Figure 4A). In contrast, 1 µg/mL APS7-2 did not completely block such an increase in intracellular calcium levels (Figure 4A).

### 2.5. Modulation of Cisplatin-Induced Cytotoxicity in A549 Cells by Nicotine, APS8-2, or APS7-2

**Figure 5:**
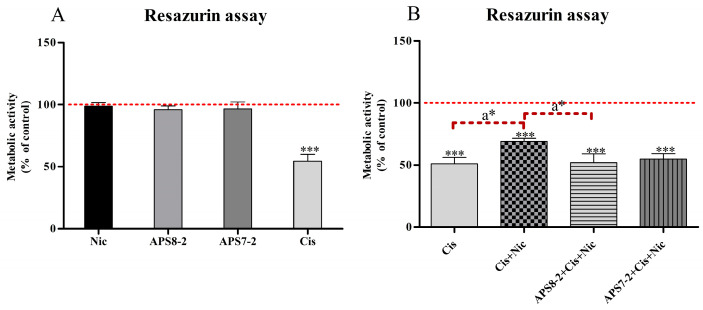
Effects of nicotine, APS7-2, APS8-2, and cisplatin on the cell viability of A549 cells. (**A**) Viability of A549 cancer cells treated with 1 µM nicotine (Nic), 1 µg/mL APS8-2, 0.5 µg/mL APS7-2, and 50 µg/mL cisplatin (Cis). (**B**) Viability of A549 cancer cells treated with 50 µg/mL cisplatin (Cis), 50 µg/mL cisplatin (Cis) + 1 µM nicotine (Nic), 50 µg/mL cisplatin (Cis) + 1 µg/mL APS8-2 + 1 µM nicotine (Nic), and 50 µg/mL cisplatin (Cis) + 0.5 µg/mL APS7-2 + 1 µM nicotine. The viability of A549 cells was measured using the RUZ assay after 24 h of treatment. Measurements were normalized to the untreated control (dashed line) as the mean percentage (±SD). Data were statistically analyzed using ANOVA with multiple comparisons and Bonferroni post-test. The asterisks indicate a significant difference compared to the untreated control (*** corresponds to *p* < 0.001) and compared to the cells treated with the combination of cisplatin and nicotine (a* corresponds to *p* < 0.05).

The effects of different treatments on the viability of A549 cells were evaluated after 24 h of treatment using the RUZ assay (Figure 5). Treatment of A549 cells with 1 µM nicotine, 1 µg/mL APS8-2, or 0.5 µg/mL APS7-2 had no effect on the viability of A549 cells (Figure 5A), while 50 µg/mL cisplatin induced cytotoxicity and significantly decreased the viability of A549 cells (Figure 5B). When the cells were simultaneously treated with 1 µM nicotine and 50 µg/mL cisplatin, nicotine significantly reduced the cytotoxicity of cisplatin (Figure 3B). Furthermore, when cells were simultaneously treated with the combination of cisplatin, nicotine, and APS8-2 or APS7-2, the cytotoxicity of cisplatin was significantly restored by APS8-2, whereas APS7-2 did not fully restore cisplatin-induced cytotoxicity (Figure 5B).

### 2.6. Modulation of Cisplatin-Induced Reactive Oxygen Species in A549 Cells by Nicotine, APS8-2, or APS7-2

**Figure 6:**
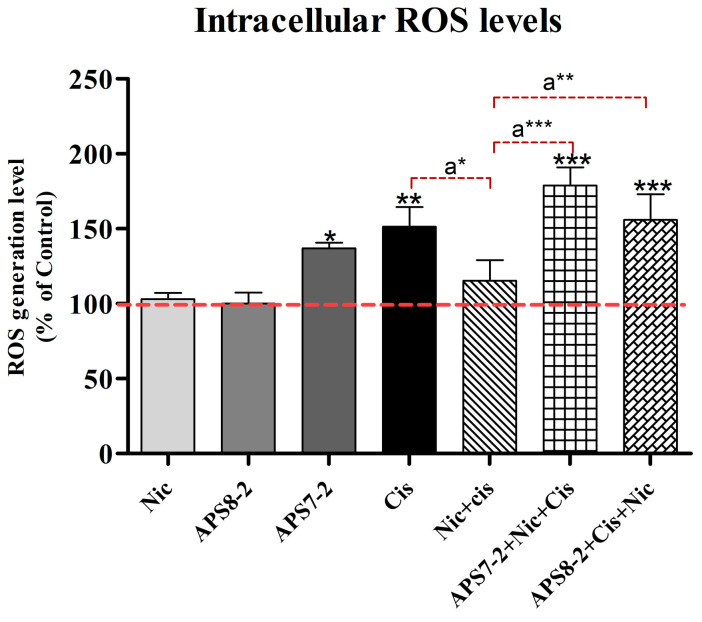
Intracellular ROS levels in A549 cells. Cells were treated with 1 µM nicotine (Nic), 1 µg/mL APS8-2, 0.5 µg/mL APS7-2, 100 µg/mL cisplatin (Cis), a combination of Nic and Cis, a combination of APS8-2, Cis, and Nic, or a combination of APS7-2, Nic, and Cis. Results were normalized to the untreated control cells (dashed line) and are presented as mean percentage (±SD). The data were statistically analyzed by ANOVA with Bonferroni multiple comparisons post-test. The asterisks indicate a significant difference compared to the untreated control: * equals *p* < 0.05; ** equals *p* < 0.01; *** equals *p* < 0.001; and with respect to cells treated with Cis and Nic, a* equals *p* < 0.05; a** equals *p* < 0.01; a*** equals *p* < 0.001.

The intracellular level of reactive oxygen species (ROS) was assessed in A549 cells following a 24 h exposure to various compounds (refer to Figure 6). Treatment with nicotine and APS8-2 did not increase ROS levels in A549 cells (Figure 6), whereas treatment with APS7-2 significantly elevated ROS levels in A549 cells (Figure 6). Upon the addition of 100 µg/mL cisplatin, ROS levels were significantly elevated in A549 cells. In cells pretreated with 1 µM nicotine, the ROS induced by cisplatin were significantly lower compared to cells that were not pretreated with nicotine. This effect of nicotine was attenuated by 1 µg/mL APS8-2 and 0.5 µg/mL APS7-2 in cells pretreated with these compounds (Figure 6).

## 3. Discussion

The activation of α7 nAChR by nicotine triggers events leading to increased cell proliferation, accompanied by elevated expression levels of α7 nAChR in lung cancer cells [21]. Recognizing the role of α7 nAChR in cancer, the idea of using α7 nAChR antagonists as potential therapies in lung cancer treatment might represent an attractive approach. In this study, we present new α7 nAChR antagonists, suggesting that nAChR antagonists could potentially be therapeutic in lung cancer treatment. Our electrophysiological measurements revealed that APS7-2 and APS8-2 possess the capability to inhibit ACh-evoked currents mediated by the α7 nAChRs subtype (Figure 1), establishing them as α7 nAChR antagonists. To assess their antagonistic impact on lung cancer development through nAChRs, their potential to inhibit nicotine-mediated effects on the proliferation and viability of A549 human lung cancer cells was investigated (Figure 3). A549 cells were specifically chosen in this study for their high expression level of α7 nAChRs [22]. Certain nAChR subtypes exhibit selective overexpression in different cancer cells. For instance, α7 nAChR is notably overexpressed in lung cancer, whereas α9 nAChRs exhibit increased expression in breast cancer [23], while both α7 and α9 containing nAChRs promote the growth of non-small-cell lung carcinoma cells [10]. Further investigation should be conducted to establish the selectivity profile of APS7-2 and APS8-2 at different human nAChR subtypes expressed in *Xenopus laevis* oocytes.

Organic synthesis can be used to produce various poly-APS analogs with defined molecular mass in order to make them more suitable for commercial production and medical application [24]. For example, APS7-2 and APS8-2, used in this study, were chemically synthesized [24], showing distinct cytotoxic responses in A549 cancer cells (Figure 2). APS7-2 exhibited a concentration-dependent cytotoxic effect in A549 cells (Figure 2B). In contrast, APS8-2 showed no cytotoxicity on A549 cells (Figure 2A). The divergence in cytotoxicity of APS7-2 and APS8-2 might be attributed to the variations in alkyl chain lengths and the presence of distinct counter ions within their chemical structures (specifically, Br- in APS7-2 and Cl- in APS8-2, respectively) (Figure 6). This underscores the significance of the chemical structure in determining the cytotoxic potential of these compounds toward cancer cells, aligning with established principles in previous research [25].

Nicotine activates α7 nAChR, leading to increased cancer cell proliferation [21]. In this study, nicotine increased the viability and proliferation of A549 cells (Figure 3). This increase in cell viability and proliferation was attenuated by using a non-cytotoxic concentration of both α7 nAChR antagonists—APS7-2 and APS8-2 (Figure 3). Similarly, nAChR antagonists such as sinomenine and QND7 were found to reduce cell proliferation and migration while enhancing apoptosis in NSCLC [26,27]. Another poly-APS analog, APS8, which shares a similar structure to APS7-2 and APS8-2, was identified as a potent α7 nAChR antagonist even at low concentrations (1–3 nM) [11]. APS8 counteracts the anti-apoptotic effects of nicotine and suppresses lung cancer cell growth with no obvious effect on normal lung fibroblasts [11]. In conclusion, our findings, in conjunction with previous studies on α7 nAChR ligands like sinomenine, QND7, and APS8, emphasize the potential therapeutic efficacy of APS7-2 and APS8-2 in counteracting the pro-cancer effects of nicotine, such as increased cancer cell viability and proliferation, offering a promising pathway in lung cancer treatment.

In addition to the role of the α7 nAChR subtype in increasing the viability and proliferation of cancer cells, this subtype is known for its Ca^2+^ permeability. The activation of these receptors by nicotine has been shown to result in an elevated influx of Ca^2+^ ions in A549 cells [28,29]. Consistent with prior studies [28,29], our findings demonstrated a significant increase in Ca^2+^ cytosolic concentration in A549 cancer cells following nicotine exposure (Figure 4). This nicotine-induced increase in Ca^2+^ ion influx was significantly prevented when cells were pretreated with APS8-2 and APS7-2, albeit to a lesser extent by the latter (Figure 4). APS8-2 also showed higher potency for inhibition of the ACh-elicited current than APS7-2 in electrophysiological measurements (Figure 1). Therefore, APS8-2 might have a higher affinity for α7 nAChR than APS7-2, or nicotine might have a higher preference for the α7 nAChR than APS7-2, resulting in an increase in cytosol Ca^2+^ concentration in cells pretreated with APS7-2 (Figure 3). Further studies should explore the binding affinity of these compounds to the α7 nAChR, comparing them with nicotine.

Nicotine exhibited no effects on the viability of A549 cells when treated in a fully supplemented medium (Figure 5). In contrast, previous studies [11,30] have shown a significant increase in the viability and proliferation of SKMES-1 and A549 lung cancer cells induced by nicotine. However, nicotine increased the viability and proliferation of A549 cancer cells treated in a serum-free medium (Figure 3). Similarly, many studies reported that nicotine increased the viability and proliferation of A549 cells in starvation conditions [10,31,32]. Kyte and Gewirtz (2018) explained that the use of a serum-free medium eliminates exogenous growth factors from the cellular medium and induces quiescence, thereby synchronizing the cell cycle [33]. Further research should be conducted to investigate the mechanisms by which nicotine induces the proliferation and viability of lung cancer cells in starvation conditions only.

A549 cells are known to develop resistance to cisplatin, a widely utilized chemotherapy drug in lung cancer treatment [6,7]. Our study showed that nicotine significantly decreased the cytotoxicity of cisplatin in A549 cells (Figure 5B). These results align with a previous study suggesting that nicotine decreases the cytotoxicity of cisplatin in oral cancer cells via activation of α7 nAChRs [12]. Therefore, nicotine might decrease the cytotoxicity of cisplatin in A549 cells via α7 nAChRs (Figure 5B). Furthermore, blocking these receptors with α7 nAChR antagonists, such as APS8, was found to reverse the effects of nicotine and disrupt cisplatin resistance in A549 cells [11]. In our study, APS8-2, which shares a similar structure with APS8, exhibited a comparable effect in counteracting nicotine effects (Figure 3) and restoring cisplatin cytotoxicity (Figure 5B), whereas APS7-2 did not fully restore cisplatin-induced cytotoxicity (Figure 5B). This result is expected as APS7-2 also did not completely block nicotine-induced increase in intracellular calcium levels in A549 cells (Figure 4). This study enhances our understanding and development of safer therapeutic agents when compared to APS8. APS8 was found to be cytotoxic on A549 cells at a concentration of 0.5 µg/mL [11], whereas APS8-2 exhibited no cytotoxic effects even at high concentrations of up to 100 µg/mL, as shown in Figure 2A.

Cisplatin-induced cytotoxicity was linked to the generation of mitochondrial ROS [34]. In this study, it was observed that nicotine decreased the intracellular ROS levels caused by cisplatin in A549 cells (Figure 6). Similarly, a previous study showed that the activation of nAChRs by nicotine can reduce oxidative stress by decreasing ROS generation and activating cellular antioxidant defenses [35]. We speculate that nicotine decreased cisplatin-induced ROS production (Figure 6) via nAChR activation, leading to a reduction in cisplatin cytotoxicity (Figure 5). Additionally, this study showed that APS8-2 counteracted the nicotine-induced decrease in ROS levels caused by cisplatin (Figure 6). This decrease in ROS was also prevented by APS7-2, although it might be attributed to ROS generated solely from APS7-2 (Figure 6). APS8-2 might bind to and block α7 nA-ChRs, thereby attenuating the effects of nicotine on cisplatin treatment. This suggests that APS8-2 offers a potential therapeutic strategy for the development of chemotherapeutic agents for lung cancer treatment.

## 4. Materials and Methods

### 4.1. Chemicals

**Figure 7:**
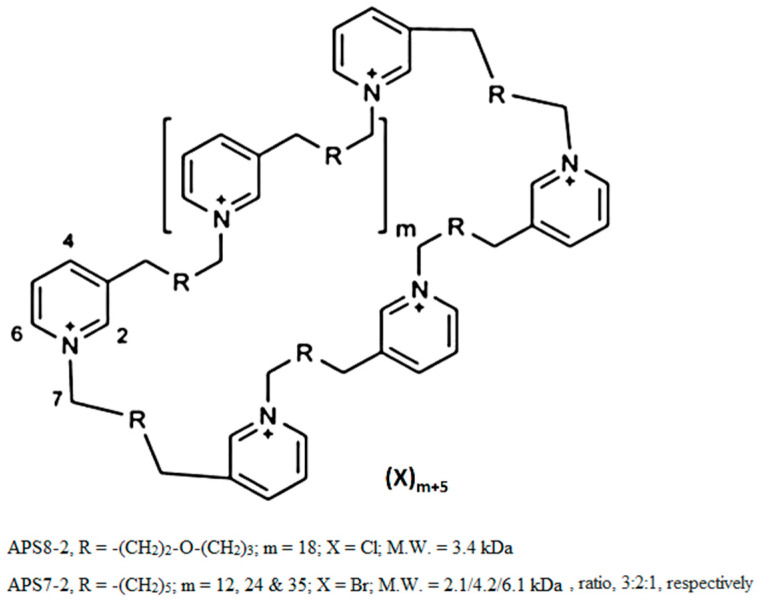
The structure of 3-alkylpyridinium salts analogs used in this study, APS7-2 and APS8-2.

APS8-2 and APS7-2 were obtained from Prof. Michael Jaspars from the University of Aberdeen, Scotland, where they were synthesized (Figure 7) [24]. A549 cells were obtained from the American Type Culture Collection (ATCC, Manassas, VA, USA). Ca^2+^ indicator Fluo-4 AM was obtained from Invitrogen (Carlsbad, CA, USA). Cell culture media, bovine serum albumin (BSA), phosphate-buffered saline (PBS), and all other chemicals used in our experiments were obtained from Sigma-Aldrich (Steinheim, Germany) unless stated otherwise.

### 4.2. Cell Culture

A549 were cultured in fully supplemented Dulbecco’s modified Eagle’s medium (DMEM), supplemented with 4 mM L-glutamine and 10% (*m*/*v*) fetal bovine serum (FBS). Cells were grown at 37 °C in a humidified atmosphere with 5% CO_2_. Prior to experimental work, cells were confirmed to be mycoplasma negative using the MycoAlertTM Kit (Lonza, Basel, Switzerland).

### 4.3. Expression of nAChRs Xenopus Laevis Oocytes and Electrophysiological Recordings

The linearized plasmids were transcribed into RNA using the T7 mMESSAGEmMACHINE transcription kit (Ambion, Austin, TX, USA) in order to express human α7 nAChRs in *X. laevis* oocytes. We obtained mature female animals from Xenopus1 (Dexter, MI, USA) and maintained them in accordance with the European Union’s (EU) regulations on laboratory animal welfare, as outlined in Directive 2010/63/EU. The use of *X. laevis* oocytes was approved by the Animal Ethics Committee of KU Leuven under license number P186/2019. Stage V–VI oocytes were harvested from females, as described previously 20. The frogs were anesthetized using a 0.1% tricaine solution (amino benzoic acid ethyl ester; Merck, Kenilworth, NJ, USA). Oocyte microinjection was carried out with a microinjector from Drummond Scientific^®^ (Broomall, PA, USA) using a programmed cRNA injection volume of 50 nL. The injected RNAs had concentrations ranging from 700 to 1500 ng/µL. The oocytes were incubated at 16 °C in ND96 solution (96 mM NaCl, 2 mM KCl, 1.8 mM CaCl_2_, 2 mM MgCl_2_, and 5 mM HEPES; pH 7.4), which was supplemented with 50 mg/L gentamicin sulfate (Panpharma GmbH, Hameln, Germany) and 180 mg/L theophylline (Sigma-Aldrich, St Louis, MO, USA).

Electrophysiological measurements were conducted at room temperature (18–22 °C) using the two-electrode voltage-clamp (TEVC) technique 2–5 days post-injection. Data were sampled at 100 Hz and low-pass filtered at 20 Hz using a four-pole Bessel filter, with a GeneClamp 500 amplifier (Axon Instruments^®^, Burlingame, CA, USA), and Clampex10 software (Axon Instruments^®^, Burlingame, CA, USA). Glass micropipettes were created from borosilicate glass capillaries (WPI 1B120-6) and drawn using a manual stretcher from WPI (World Precision Instruments^®^, Sarasota, FL, USA). We used previously described ND96 for the bath and perfusion solution.

Cells were clamped at a holding potential of −70 mV and continuously perfused with ND96 buffer. For α7 nAChR measurements, current responses were elicited by applying 100 µM ACh (Sigma-Aldrich, St Louis, MO, USA) dissolved in ND96 buffer under gravitational flow until peak current amplitude was observed. A control consisting of 3 pulses of ACh was conducted, with a 30 s interval between each pulse. APS7-2 and APS8-2 were applied directly in the perfusion chamber and incubated for 2 min without gravitational flow. Subsequently, a new ACh pulse was administered. Peak current amplitudes were measured both before and after compound incubation. The concentration of 100 μM ACh used represents the EC_50_ (half-maximal effective concentration) for the human α7 nAChR subtype.

To assess the concentration–response relationships, data points with standard error of the mean (SEM) were fitted with the Hill equation:

y = 100/[1 + (IC_50_/[toxin])h]

where y is the amplitude of the toxin-induced effect, IC_50_ is the toxin concentration at half-maximal efficacy, [toxin] is the toxin concentration, and h is the Hill coefficient. Each data point on the curve is presented as the mean ± standard error (SEM) of at least six independent experiments (*n* ≥ 6).

### 4.4. Cell Viability and Proliferation Measurements

#### 4.4.1. Cytotoxicity of APS8-2 and APS7-2

The cytotoxicity of APS8-2 and APS7-2 on A549 lung cancer cells for 24 h exposure was assessed using the resazurin assay (RUZ), neutral red uptake (NRU) assay, and Coomassie Brilliant Blue (CBB) assay. RUZ and NRU assays were used to measure cell viability by measuring the metabolic activity of cells and accumulation of the neutral red dye in the lysosomes of live cells, respectively, while the CBB assay was used to measure cell proliferation by measuring the amount of cellular protein in live cells. A549 cells were seeded into a 96-well plate at a seeding density of 7000 cells/well in fully supplemented DMEM. After 24 h incubation, A549 cells were treated with APS7-2 (0.25–10 µg/mL) and APS8-2 (0.5–10 µg/mL). RUZ, NRU, and CBB measurements were performed as described by Kononenko and Drobne (2019) [36]. Briefly, the cells were stained and measured using a spectrofluorimeter (BioTek, Cytation 3). At least three independent experimental repeats, each with five replicates, were performed for all treatments.

#### 4.4.2. Modulation of Cisplatin-Induced Cytotoxicity in A549 Cells by Nicotine, APS8-2, or APS7-2

A549 cells were seeded into 96-well plates at a seeding density of 7000 cells/well in fully supplemented DMEM. Cells were treated with APS8-2 (1 µg/mL), APS7-2 (0.5 µg/mL), cisplatin (50 µg/mL), and nicotine (1 µM), as well as the combination of cisplatin (50 µg/mL) and nicotine (1 µM), the combination of cisplatin (50 µg/mL), nicotine (1 µM), and APS8-2 (1 µg/mL), and the combination of cisplatin (50 µg/mL), nicotine (1 µM), and APS7-2 (0.5 µg/mL) for 24 h. RUZ assay was performed as described by Kononenko and Drobne (2019) [36]. Briefly, the cells were stained and measured using a spectrofluorimeter (BioTek, Cytation 3). At least three independent experimental repeats, each with five replicates, were performed for all treatments.

#### 4.4.3. Attenuation of Nicotine-Induced Increased Cell Viability and Proliferation by APS8-2 and APS7-2

A549 cells were seeded into 96-well plates at a density of 3000 cells/well in serum-deprived cell medium for 24 h, followed by 1 µM nicotine treatment in serum-deprived cell medium for another 24 h. The cell medium was then changed to a fully supplemented medium with 0.5 µg/mL APS8-2 or APS7-2 for 24 h. Thereafter, measurements were performed with RUZ and CB assays, as described previously by Kononenko and Drobne (2019) [36]. For each treatment condition, three independent experimental repeats, each with at least five replicates, were performed.

### 4.5. Calcium Imaging

The modulation of intracellular Ca^2+^ levels in A549 cells by nicotine, APS8-2, and APS7-2 was measured by Ca^2+^ indicator Fluo-4 AM and time-lapse microscopic imaging. A549 cells were seeded at a density of 80,000 cells/well in 12-well plates with inserted sterile coverslips for 24 h in serum-deprived cell culture medium. Then, cells were pretreated with 1 µg/mL APS7-2 or 1 µg/mL APS8-2 for another 24 h. Adhered cells on coverslips were stained by 2.5 µM Fluo-4 AM for 30 min according to the manufacturer’s recommendations. After that, the coverslips were washed three times with Hank’s balanced salt solution (HBSS), and then the coverslips were left for 30 min in HBSS. A549 cells were monitored for changes in intracellular calcium ion concentrations caused by the addition of 1 mM of nicotine. Time-lapse images of A549 cells were acquired at 5 s intervals using fluorescence microscopy (Axio Imager.Z1; Carl Zeiss, Jena, Germany). Changes in Fluo-4 fluorescence intensity in A549 cells were determined using ImageJ software (National Institutes of Health, Bethesda, MD, USA) for the first 100 s after adding nicotine. For each treatment condition, at least three technical repetitions were performed where the fluorescence of at least 30 individual cells was evaluated.

### 4.6. Intracellular ROS Measurement

A549 cells were seeded in 96-well plates at a density of 7000 cells per well and allowed to adhere for 24 h. Following adhesion, the cells were treated for 24 h with various compounds: 1 µM nicotine, 1 µg/mL APS8-2, 0.5 µg/mL APS7-2, and combinations of these compounds with 1 µM nicotine. After treatment, cells were washed with 100 µL of PBS. Subsequently, the cells were exposed to 20 µM 2′,7′-dichlorofluorescein diacetate (DCFH-DA) for 30 min to load the ROS indicator, followed by two PBS washes to remove excess dye. Finally, the cells were treated with 100 µg/mL cisplatin, and the fluorescence of 7′-dichlorofluorescein (DCF) was measured using a BioTek Cytation 3 microplate reader (USA) at an excitation wavelength of 488 nm and an emission wavelength of 520 nm to assess intracellular ROS levels. The compound undergoes oxidation induced by ROS, resulting in the formation of highly fluorescent DCF. Each treatment condition was repeated independently at least three times.

### 4.7. Statistical Analysis

The results of cytotoxicity, viability, and proliferation measurements, as well as calcium imaging, are presented as the mean ± standard deviation (SD) and statistically analyzed using ANOVA with Bonferroni post-test for multiple comparisons. Statistical significance was determined by a *p*-value below 0.05. The analyses were conducted using GraphPad Prism software (GraphPad Software, San Diego, CA, USA). Statistical analysis of channel activity differences between the control and toxin conditions was performed using a one-way ANOVA, followed by Tukey’s multiple comparisons test, with GraphPad Prism software. The IC50 values are presented ± SEM (standard error of the mean). Statistical significance was considered when *p* < 0.1.

## 5. Conclusions

The cytotoxicity of synthetic APS compounds is linked to their chemical structure. The impact of nicotine on lung cancer viability and proliferation is more pronounced under cellular starvation conditions. This study demonstrates the potential therapeutic efficacy of new α7 nAChR antagonists, APS7-2 and APS8-2, in countering the pro-cancer effects of nicotine on A549 human lung cancer cells. We conclude that APS8-2 is a promising new therapeutic agent in the chemotherapy of lung cancer.

## Data Availability

The data supporting the findings of this study are available from the corresponding author upon request.
